# An interdisciplinary intervention for health prevention and promotion in a Roman neighborhood

**DOI:** 10.1093/eurpub/ckac130.139

**Published:** 2022-10-25

**Authors:** A Lontano, C De Waure, E Marziali, F D'Ambrosio, C Galletti, E Mazza, A Mingarelli, E Urbani, V Galasso, P Laurenti

**Affiliations:** 1 Hygene and Preventive Medicine, Università Cattolica del Sacro Cuore, Rome, Italy; 2 Università degli Studi di Perugia, Perugia, Italy; 3 Fondazione Policlinico Universitario A. Gemelli, Rome, Italy; 4 Azienda Ospedaliera San Camillo Forlanini, Rome, Italy; 5 DiagnostiCare ONLUS, Rome, Italy; 6 Sapienza Università di Roma, Rome, Italy

## Abstract

Influencing behavioral patterns through primary prevention, possibly addressing more risk factors at a time, is the most effective means to tackle cardiovascular diseases. Many interdisciplinary prevention activities have been coordinated by community nurses outside of specialist centers, resulting in a more effective control of risk factors. Our study aims at describing the impact of an 18-month prevention and promotion, interdisciplinary intervention on lifestyle habits and cardiovascular risk. From December 2018 to May 2020, patients were recruited by 4 General Practitioners (GPs) in the Roman neighborhood of Torresina and received nutritional, physical and psychological counselling to learn healthy lifestyles. Until May 2020 patients had to self-manage their new healthy habits, but during this phase the SARS-CoV-2 pandemic broke out. Patients were assessed at baseline, 6, 12 and 18 months by a nutritionist, a physiotherapist, a psychologist, the 4 GPs and community nurses, and the cardiovascular risk score (CRS) was estimated at every examination. 76 patients were included, with a mean age of 54,6 years. Mean CRS showed a significant reduction between baseline and 12 months (from 4.9 to 3.8, p < 0.001), but this trend was not maintained at 18 months. As for variables included in the calculation of the cardiovascular risk score, both total cholesterol and systolic blood pressure significantly decreased at 6 months of follow up (respectively, from 211.1 to 192 (p < 0.001) and from 133.1 to 123.1(p < 0.001)). Nontheless, the reduction was maintained in the remaining points in time only for systolic blood pressure. Our interdisciplinary educational intervention in a primary care setting resulted in a CRS improvement at 12 months, but this changes where not maintained at 18 months. Community nurses were facilitators in improving health outcomes and patient's satisfaction in the described primary care setting.

**Key messages:**

- Our interdisciplinary educational intervention in a primary care setting resulted in a CRS improvement at 12 months, but this changes where not maintained at 18 months.
- Community nurses are facilitators in improving health outcomes and patient’s satisfaction in the described primary care setting.

